# A critical reflection on definitions of autistic well‐being

**DOI:** 10.1002/brb3.3572

**Published:** 2024-06-30

**Authors:** M. Deakin, S. Petty, B. Heasman, L. G. Hamilton

**Affiliations:** ^1^ School of Education, Language and Psychology York St John University York UK

**Keywords:** autism, neurodiversity, well‐being

## 1 A CRITICAL REFLECTION ON DEFINITIONS OF AUTISTIC WELL‐BEING

Our ongoing systematic literature review of psychological well‐being interventions for autistic adults highlighted that the concept of well‐being has been poorly operationalized for autistic people in clinical literature. Our investigation aims to contribute a novel review of how well psychological well‐being interventions are designed for autistic adults. While assumptions around the definition of autism have been critiqued extensively in literature (Heasman & Parfitt, 2023) our review of 69 studies found that the concept of “well‐being”, as it applies to autistic people, has received comparatively little attention. We raise three issues with the current oversimplification of well‐being when it is understood as a return to “normal” functioning through the remediation of ill‐being. We suggest that a way forward is to embrace contemporary theorizing of both being autistic (Chapman, 2021; Kourti, 2021) and of experiencing wellness vulnerabilities (van Os et al., 2019). A review of what is meant by autistic well‐being would inform clinical and research practices.

## 2 REPRESENTATION OF AUTISTIC WELL‐BEING

In our review we have found that representations of well‐being of autistic people were diverse; however, they lacked critical evaluation. Well‐being was operationalized as a wide range of outcomes, such as a reduction in anxious thoughts (Langdon et al., 2016), or an increase in social relating (Oswald et al., 2018). Some studies measured changes in multiple domains, such as stress, anxiety, depression and quality of life (Pahnke et al., 2019). Importantly, many authors either (a) commented that the outcome measures used to demonstrate the effectiveness of a therapeutic intervention were not designed for autistic people or (b) did not specify how the measures would be appropriate for use with autistic people. This lack of criticality can lead to misinterpretations (Stewart et al., 2006). For example, an autistic person might reduce social demands temporarily as a way to improve their well‐being, rather than this being a reflection of ill‐being (Pearson & Rose, 2021). This highlights how autistic people have described their distress being misunderstood in clinical practice (Au‐Yeung et al., 2019; Babb et al., 2021), emphasizing the need to hear lived experiences over default definitions. Systematic reviews have identified wide variability in the reported rates of anxiety, depression and suicide ideation for autistic people (Cassidy et al., 2018; Hollocks et al., 2019; Wigham et al., 2017). For example, one review identified that rates of depression reported for autistic people without cognitive impairment varied from 1% to 47% (Wigham et al., 2017). Potential reasons for this variation include the lack of definitive conceptualizations of depression for autistic people, as well as uncertain validity of the outcome measures used. One response to these concerns has been to develop measures designed with and for autistic adults, for example the *Anxiety Scale for Autism—Adults* (Rodgers et al., 2020). Together, these findings show the need for a stable definition of “autistic well‐being” to aid further development of appropriate tools for research and clinical practice.

## 3 WELL‐BEING MAY BE DIFFERENT FOR AUTISTIC PEOPLE

The experience of well‐being may be different for autistic people compared with their non‐autistic peers, but measures of autistic well‐being are often based on normative models (Ayres et al., 2018; Lam et al., 2021). Assuming that being well means being “typical” is counterproductive (Milton & Sims, 2016). Current guidance tells practitioners to follow established therapeutic intervention protocols for the general population, and to tailor these for autistic people (NICE, 2021; Scottish Intercollegiate Guidelines Network, 2016). A critical review of the conceptualization of autistic well‐being will open up research possibilities. Many studies featured in our ongoing review were exploratory pilot or feasibility studies, reflecting the early development of research in this area. Developing work is considering concepts of marginalization and societal justice more broadly (Petty, Hamilton et al., 2023). There are associated implications for individual well‐being, including an autistic person's sense of belonging (Milton & Sims, 2016; Sonuga‐Barke, 2023).

To direct future research, it is helpful to note that the challenge of identifying a stable definition of well‐being is not limited to autistic people. Dodge et al. (2012) defined well‐being as “the balance point between an individual's resource pool and the challenges faced, where resources and challenges can be psychological, social and physical,” (p. 230). However, well‐being is more than an absence of illness or negative stressors (Dodge et al., 2012; Waters et al., 2009). The idea that ill‐being inevitably stems from being autistic needs to be challenged; instead ill‐being stems from a combination of external and internal factors (Chapman, 2020). A revised understanding of autistic well‐being will need to consider elements often missed from definitions created for a neurotypical majority, including sensory processing differences (Milton & Sims, 2016; Petty, Lambarth et al., 2023; Robeyns, 2016). Sensory processing is a dimension of well‐being that can help understand pain and fatigue for autistic people (Robeyns, 2016). A scale of overwhelm or fatigue may offer a better metric of emotional distress than standardized tools assessing anxiety or depression (Petty, Lambarth et al., 2023). A stronger conceptualization of autistic well‐being requires a theoretical rationale about what well‐being is, and a nuanced approach to how this interacts with characteristics of being autistic, intersectional complexities, and support needs. This conceptualization would appreciate how well‐being is situated within societal and structural environments that may support or limit well‐being capabilities (Lam et al., 2021; Robeyns, 2016). Importantly, the potential to flourish also needs to be recognized. Chapman and Carel (2022) write about the implications of epistemic injustice that denies autistic flourishing, and can mislead some autistic individuals’ attempts to thrive. We recommend a way of conceptualizing well‐being to underpin meaningful measures that fairly represent autistic people's various diverse experiences, rather than “adding to” concepts developed for the general population.

## 4 RECONCEPTUALIZED AUTISTIC WELL‐BEING

Contemporary theorizing provides the opportunity to refocus the purpose of therapeutic interventions, disentangle the need to reduce problematic “symptoms” alone, and promote thriving (Sonuga‐Barke, 2023; van Os et al., 2019). Our mixed‐methods review had deliberately broad inclusion criteria for psychological interventions, outcomes and study designs. Yet the studies reviewed predominantly focused on reducing symptoms of ill‐being. Alternatively, mental health can be conceptualized as a person's vulnerabilities, within a resilience‐building approach that would strengthen social and existential domains (van Os et al., 2019). Having a positive autistic social identity appears to offer a protective mechanism for psychological well‐being through improved personal and collective self‐esteem (Cooper et al., 2017).

In conclusion, we recommend that the ontological reframing of autism flows through into clinical practice. We suggest that emerging research into therapeutic interventions for autistic people foregrounds a valid well‐being definition and personally important lifespan outcomes, without comparison with a neuromajority default. This approach would align with a neurodiversity paradigm model for translational science (Sonuga‐Barke, 2023).

The authors have clinical and research experience of working with autistic clients, and align with a neurodiversity approach that values differences (Dwyer, 2022). They are not diagnosed as autistic.

## AUTHOR CONTRIBUTIONS

**Michele Deakin**: Conceptualization; writing—original draft; writing—review and editing. **Stephanie Petty**: Conceptualization; writing—original draft; writing—review and editing. **Brett Heasman**: Conceptualization; writing—original draft; writing—review and editing. **Lorna G Hamilton**: Conceptualization; writing—original draft; writing—review and editing.

## CONFLICT OF INTEREST STATEMENT

The authors have no conflicts of interest to disclose.

### PEER REVIEW

The peer review history for this article is available at https://publons.com/publon/10.1002/brb3.3572.

## Data Availability

The data that support the findings of this study are available from the corresponding author upon reasonable request.
